# The Impact of Automated Brief Messages Promoting Lifestyle Changes Delivered Via Mobile Devices to People with Type 2 Diabetes: A Systematic Literature Review and Meta-Analysis of Controlled Trials

**DOI:** 10.2196/jmir.5425

**Published:** 2016-04-19

**Authors:** Carukshi Arambepola, Ignacio Ricci-Cabello, Pavithra Manikavasagam, Nia Roberts, David P French, Andrew Farmer

**Affiliations:** ^1^ University of Colombo Faculty of Medicine Colombo Sri Lanka; ^2^ University of Oxford Nuffield Department of Primary Care Health Sciences Oxford United Kingdom; ^3^ University of Oxford Bodleian Libraries Oxford United Kingdom; ^4^ University of Manchester School of Psychological Sciences Manchester United Kingdom

**Keywords:** Diabetes mellitus, type 2, mobile health, text messaging, systematic review, diet, physical activity, self-care

## Abstract

**Background:**

Brief automated messages have the potential to support self-management in people with type 2 diabetes, but their effect compared with usual care is unclear.

**Objective:**

To examine the effectiveness of interventions to change lifestyle behavior delivered via automated brief messaging in patients with type 2 diabetes.

**Methods:**

A systematic literature review of controlled trials examined the impact of interventions, delivered by brief messaging, and intended to promote lifestyle change in people with type 2 diabetes, on behavioral and clinical outcomes. Bibliographic databases searched included Medline, Embase, CINAHL, PsycINFO, and ISI WoK. Two reviewers independently screened citations. We extracted information on study risk of bias, setting (high versus low- and middle-income countries) and intervention characteristics (including use of theory and behavior-change techniques). Outcome measures included acceptability of the interventions and their impact on 1) determinants of lifestyle behavior (knowledge about diabetes, self-efficacy, attitudes towards self-management), 2) lifestyle behavior (diet, physical activity), and 3) clinical and patient-reported outcomes. Where possible, we pooled data using random-effects meta-analyses to obtain estimates of effect size of intervention compared to usual care.

**Results:**

We identified 15 trials (15 interventions) meeting our inclusion criteria. Most interventions were delivered via short message service text messaging (n=12) and simultaneously targeted diet and physical activity (n=11). Nine interventions consisted of unidirectional messages, whereas six consisted of bidirectional messages, with patients receiving automated tailored feedback based on self-reported data. The acceptability of the interventions, and their impact on lifestyle behavior and its determinants, were examined in a low proportion of trials, with heterogeneous results being observed. In 13 trials (1155 patients) where data were available, there was a difference in glycated hemoglobin of -0.53% (95% CI -0.59% to -0.47%) between intervention groups compared to usual care. In five trials (406 patients) there was a non-significant difference in body mass index of -0.25 kg/m2 (95% CI -1.02 to 0.52). Interventions based on unidirectional messages produced similar effects in the outcomes examined, compared to those based on bidirectional messages. Interventions conducted in low- and middle-income countries showed a greater impact than those conducted in high-income countries. In general, trials were not free of bias and did not use explicit theory.

**Conclusions:**

Automated brief messages strategies can improve health outcomes in people with type 2 diabetes. Larger, methodologically robust trials are needed to confirm these positive results.

## Introduction

The number of people with type 2 diabetes worldwide is currently estimated to be 387 million, and is expected to increase to 592 million by 2035 [1]. This prevalence imposes a substantial burden of disease, mainly due to life-long multi-organ complications [2], leading to increased disability and premature deaths in low- and middle-income countries (LMIC) as well as high-income countries (HIC) [3].

Available evidence suggests that better control of blood glucose, blood pressure, and cholesterol levels would delay the onset of complications, and thereby prevent premature deaths among those already diagnosed with diabetes [4]. Lifestyle modification focusing on healthy diet is an accepted component of management [5], alongside promotion of physical activity [6]. However, patients with diabetes do not always follow advice about recommended changes in diet and physical activity, and therefore do not achieve optimal control of risk factors. Reasons for this are multifactorial, including psychological, social, and health care related factors [7,8].

Recommendations for supporting diabetes self-management are now widely incorporated in clinical practice guidelines [9,10]. However, strategies for providing effective continuing support and motivation are not well developed, and facilitating sustained behavior-changes remains an important challenge. Emerging evidence suggests that mobile health (mHealth) interventions may improve cardiovascular-related lifestyle behaviors and disease management [11]. Interventions based on brief messages delivered via mobile device technologies, such as short message service (SMS) technology, are one of the most studied types of mHealth interventions, and available evidence suggests that they may contribute to behavior-change [11,12]. Messages can be readily delivered at a wide-scale and at a low cost, and can be used to provide information through one-way (unidirectional) systems, but also to facilitate two-way communication (interactive or bidirectional). In contrast to more resource-intensive one-to-one clinician-patient contacts in clinics, the use of brief messages can be attractive for patients in terms of convenience, acceptability, and user-friendliness [13,14]. This type of intervention could address non-adherence to lifestyle recommendations by providing frequent reminders, motivational support and prompts to action, as well as timely access and feedback to relevant health information, while making patient-provider communication much easier [15]. Although their impact in different resource settings is still unclear, automated messaging technologies could be especially relevant in low-resource settings, given their ubiquity, low cost, and potential to underpin a developing health care infrastructure (eg electronic medical records systems). All of these features are leading to an increasing interest in the use of brief messages as a part of public health interventions.

A number of systematic reviews have provided evidence for the effectiveness of mobile-phone based interventions on self-management of long term conditions [11,16-19]. However, so far no study has specifically reviewed the potential impact of automated brief messages on promoting lifestyle modifications in patients with type 2 diabetes. In addition, the theoretical basis for this type of intervention is not well established, and the usefulness of basing messages on established behavior-change techniques is not known. These gaps in knowledge may partly be due to inadequate characterization of the techniques being used, which prevents the identification of those that might be helpful.

The primary objective of this systematic review was to examine the effectiveness of brief messages in improving glycemic control through promoting healthy eating and increasing physical activity, compared to usual care. We focused specifically on interventions delivered via mobile devices to people with type 2 diabetes. Secondary objectives included 1) examining the extent to which interventions have used explicit theory, 2) examining the behavior-change techniques used, 3) examining the acceptability of the interventions, 4) examining their impact on lifestyle-change behavior and its determinants, 5) examining their impact on other clinical (blood pressure, lipids, and weight) and patient-reported outcomes, 6) comparing the impact of unidirectional vs bidirectional messages, and 7) exploring the specific impact of the interventions in HIC and LMIC.

## Methods

The study was planned, conducted, and reported according to the Preferred Reporting Items for Systematic Reviews and Meta-Analyses (PRISMA) guidelines [20]. The review protocol was registered in the PROSPERO International prospective register of systematic reviews (registration number CRD42015024302).

### Data Sources and Searches

Specific search strategies were designed for the following databases (Multimedia Appendix 1): Medline, Embase, Cumulative Index of Nursing and Allied Health (CINAHL), PsycINFO, Cochrane Central Register of Controlled Trials (CENTRAL), and Science Citation Index & Conference proceedings Citation Index (ISI Web of Knowledge). To ensure the identification of relevant studies carried out in LMIC, we also searched the following databases: African Index Medicus, Index Medicus for the Eastern Mediterranean Region, Index Medicus for South-East Asia Region, Inter-Science Latin American and Caribbean Health Sciences Literature, Western Pacific Region Index Medicus, and World Health Organization Library Database (all accessed via the Global Health Library).

The search strategy combined Medical Subject Headings terms and free-text keywords (Multimedia Appendix 2). Databases were searched from inception to April 2015 and no language restriction was applied. In addition, potentially relevant studies were identified using a snowball technique initiated by the examination of 52 previous systematic reviews on the broader area of telehealth and diabetes. A bibliographical database was created using EndNote X7, which was used to store and manage the references.

### Study Selection

We included controlled trials examining the impact of interventions intended to promote lifestyle changes on diet and physical activity among people with type 2 diabetes. All interventions were delivered by brief messaging using mobile devices, and were compared on behavioral and clinical outcomes, versus usual care.

The main component of eligible interventions was the provision of information via brief messaging systems, characterized by automated messages (including computer-generated messages following an algorithm), which were tailored/custom-made personalized messages, or bulk messages. Messages had to be received via devices using mobile technology, such as mobile phones, smart-phones or hand-held computers. Messaging systems were those using the following technologies: SMS, automated email, or software apps. These systems could be used to provide information through one-way (unidirectional) messages or to facilitate two-way communication (interactive or bidirectional). Unidirectional messages were conceptualized as messages sent from the providers or researchers to the participants. Bidirectional messages were conceptualized as those involving a two-way communication regarding self-monitoring data that was sent by the participants, who in return received real-time automated brief messages providing tailored feedback. Eligible interventions targeted healthy eating, physical activity, or both.

Inclusion criteria for study design specifically focused on controlled clinical trials, including randomized controlled trials (RCTs), cluster RCTs, non-randomized controlled trials, and crossover studies. Only studies with control or comparator arms that consisted of patients receiving usual (standard) clinical care, or a minimal intervention (ie an intervention that is unlikely to produce any effect, such as sending non-health related messages, but allows blinding participants to condition allocation) were eligible for inclusion. Eligible studies included adult (at least 18 years old) patients with type 2 diabetes mellitus (with or without comorbid conditions) and had to be set in the community or in any primary, secondary, or tertiary care setting.

Studies reporting at least one of the following outcome measures were included: acceptability by recipients, determinants of change in lifestyle (namely knowledge, attitudes and self-efficacy on lifestyle modifications), lifestyle behaviors that impact on diabetic control (diet and physical activity), and clinical outcomes (glycated hemoglobin [HbA1c], body mass index [BMI] or body weight, lipids, blood pressure, and waist circumference).

Trials were excluded if they examined the use of messages created by a clinician based on a clinical judgement of a patient’s disease status (ie, not automated), had a proportion of patients with type 2 diabetes lower than 90%, evaluated a multifaceted intervention in which brief messages were not the main component of the intervention, or were published only in the form of conference abstracts. No language restrictions were applied.

A preliminary screen for eligibility was followed by retrieval and assessment of full texts of the selected articles. Studies that met the inclusion criteria were included for data extraction. All citations were independently screened by two reviewers. Any disagreements were solved by consensus with a third reviewer.

### Data Extraction and Quality Assessment

Structured forms were used to extract data about the trial design, trial setting (HIC vs LMIC according to the World Bank classification [21]), number of participants in each group, length of follow-up, key elements of the intervention, type of comparison group, and acceptability of the intervention. In addition, the impact on determinants of change in lifestyle, diet and physical activity behavior, and clinical outcomes were recorded.

The extent to which the trials used theory explicitly in the development and evaluation of the interventions was assessed using an established coding-scheme which contains 19 items. This scheme assessed whether a theory was mentioned, how theories were used in intervention design and in the selection of intervention techniques, how intervention evaluations tested theory, and the implications of the results for future theory development [22]. The behavior-change techniques used in each intervention were classified using an established taxonomy [23].

We used the Cochrane Collaboration’s tool for risk of bias assessment [24]. Two reviewers independently extracted all information and assessed the risk of bias and the use of theory. Disagreements were discussed with a third reviewer until consensus was reached.

### Data Synthesis and Analysis

We examined and synthesized the acceptability of the interventions, and their impact on 1) determinants of change in lifestyle, 2) behavior (diet and/or physical activity), and 3) clinical outcomes. Outcomes in all studies were examined and classified as measuring one of these three domains. Variables that measured other domains were not included in the analysis. For all pooled outcomes we used subgroup analyses to examine potential differences between types of messages (unidirectional vs bidirectional) and settings (HIC vs LMIC).

From each study we extracted the mean and standard deviation (SD) of HbA1c levels and BMI, contacting study authors when the information was not available. We transformed this information into weighted mean difference (95% CI), and pooled the data using random-effects models. Where SD of the change between intervention and control group for an outcome was not provided, we derived them from baseline and final SDs, assuming a correlation of 0.5 [25]. A sensitivity analysis was undertaken using different values of correlation to determine whether the overall result of the analysis was robust to the use of imputed correlation coefficients. Heterogeneity was quantified by the *I* ^2^statistic, where *I* ^2^>50% was considered evidence of substantial heterogeneity [26]. Publication bias was examined with funnel plots and presence of asymmetry tested with Begg [27] and Egger tests [28]. Meta-analyses were conducted with Stata, version 12.0. We set a threshold of *P*=0.05 to accept statistical significance.

## Results

### Trial Identification

**Figure 1:**
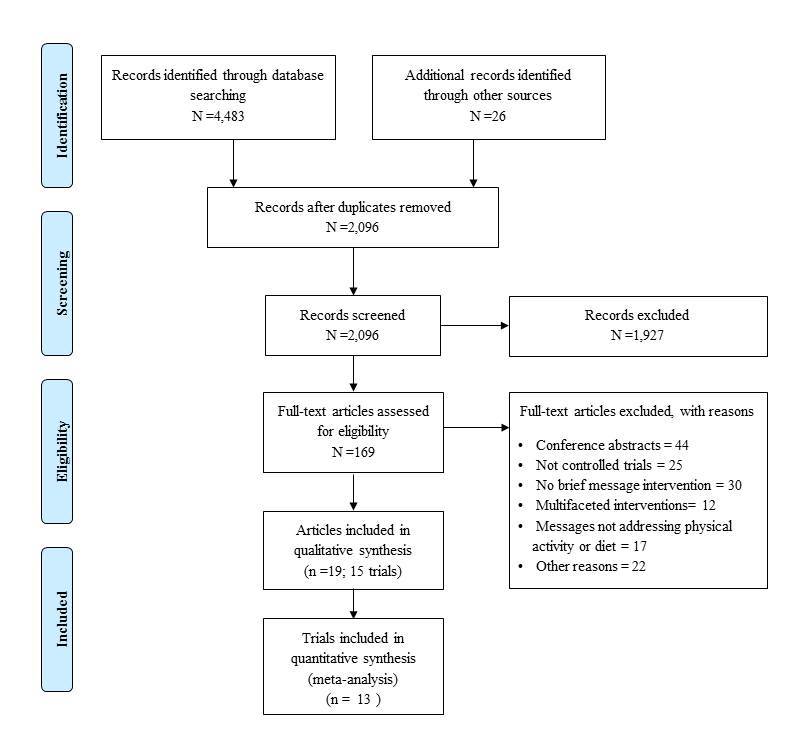
Flowchart of articles included at each stage of the screening process.

Search results are summarized in the PRISMA flow diagram (Figure 1). The initial search identified a total of 2096 unique citations. Title and abstract screening of these citations resulted in the inclusion of 169 citations for further review. Following full text screening, 19 articles [29-47] reporting on 15 separate trials (evaluating 15 separate interventions) were finally included.

### Characteristics of Trials and Interventions

Each trial assessed only one intervention. Thirteen trials used an RCT design, whereas the remaining two used a cluster-RCT [39,40] and a crossover design [44]. The average number of participants per trial was 92 (SD=52), ranging from 19 to 215. Nine of the trials were conducted in HIC, whereas six were conducted in LMIC (Multimedia Appendix 3).

On average, interventions lasted 7 months (SD=4). The majority of trials (n=12) used SMS technology to deliver the messages, whereas the rest were based on graphical information presented to the patients [44], texts available in a website [37], or brief video-messages [31]. Approximately three quarters of the interventions (n=11) addressed both diet and physical activity. Nine interventions consisted of unidirectional messages and six of bidirectional messages. Bidirectional messages were usually initiated by the study participants, who were instructed to use specific devices to conduct the glucose (and in some interventions blood pressure) measurements and transmit the results to the study database using their mobile devices. Other systems used by patients to transmit self-management information included a hand-held electronic diary that allowed participants to describe their meals by selecting the food ingredients [44], or an SMS-based system that allowed them to record their physical activity [47]. In all interventions for each reported measurement, the patient received automated, real-time educational, behavioral, and motivational messaging specific to the entered data, on the basis of a decision-support algorithm.

Twelve behavior-change techniques were used in total. Most interventions used multiple techniques. Most frequently used techniques consisted of 1) providing information about the consequences of inaction, 2) providing instructions about how to perform a behavior, and 3) providing feedback on performance – each of which were used in eight interventions. Whereas all studies examined the impact of the interventions on clinical outcomes, behavior-change was only measured in six of them, with a wide range of instruments being used.

### Risk of Bias

Only a minority of the trials presented low risk of bias, and none was completely free of bias (Multimedia Appendices 4 and 5). Most frequent biases were related to blinding of participants and personnel to the interventions (eight trials with high risk of bias), and other sources of bias (seven trials), which were mostly related to small sample sizes that did not allow the detection of clinically meaningful differences.

### Acceptability of the Interventions

Five trials examined whether unidirectional messaging interventions were acceptable to participants. Three [29,30,32,33,42] reported high acceptability and satisfaction. However, one [29,33] reported moderate usability, with 40% of the participants requesting to stop receiving the messages before the end of the intervention. Another trial, in which messages were available through a website, reported low acceptability due to the lack of a user-friendly interface and inexperience with mobile web use [37]. In a trial evaluating the use of video-messages [31] it was observed that 47% of the participants in the intervention group did not view videos at all, or did so only briefly at the beginning of their participation and then stopped in the first two months.

Four trials examined participants’ acceptability of bidirectional messaging interventions, consistently observing high acceptability in terms of easiness to use systems, usefulness, and general satisfaction [38,44,45,47].

### Effectiveness of the Interventions

#### Impact on Determinants of Behavior-Change

The impact of unidirectional messages on behavior-change determinants was only examined by two trials. One study observed significant improvement in diabetes knowledge and self-efficacy, but not in self-management attitudes [34], whereas the other observed no effect on knowledge or self-efficacy [30,32]. None of the trials evaluating bidirectional messages examined the potential impact on determinants of behavior-change (Multimedia Appendix 6).

#### Impact on Behavior-Change

Four trials examined the impact of unidirectional messages on diet and physical activity. Two studies reported no effects [30,32,42], whereas the remaining two reported statistically significant improvements in both diet and physical activity [34,43]. Only one trial examined the impact of bidirectional messages on behavior-change [45], reporting no effects.

#### Impact on Clinical Outcomes

**Figure 2:**
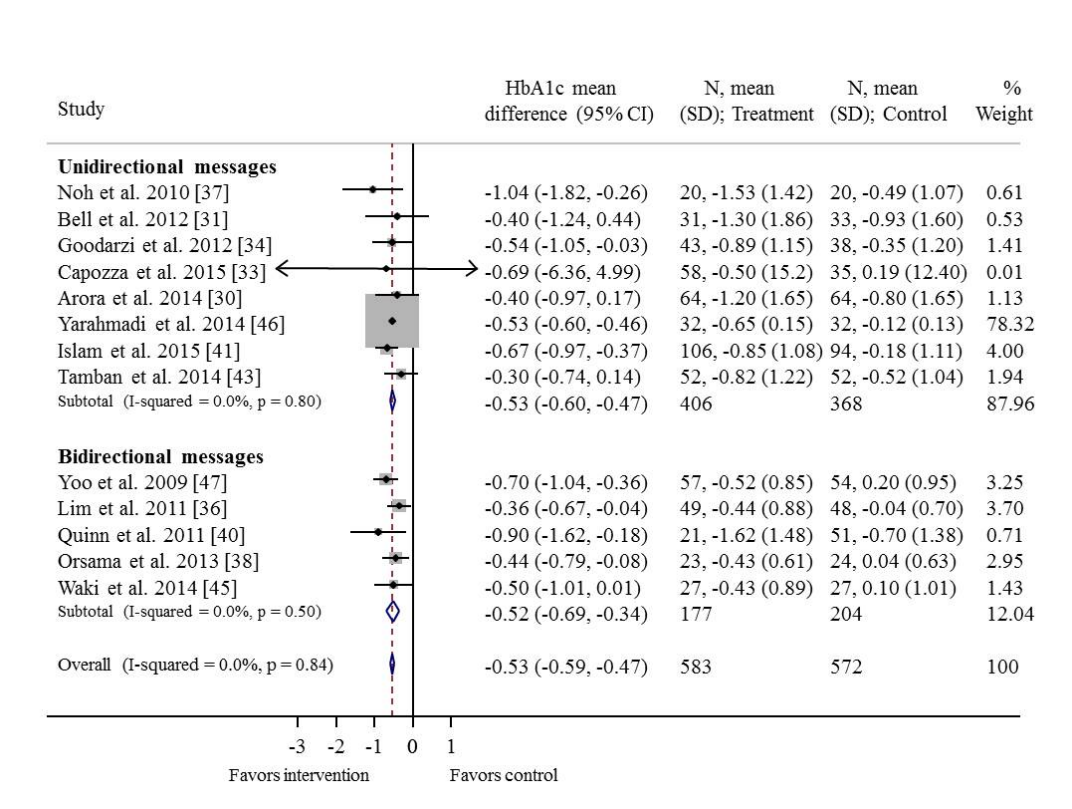
Weighted mean difference in size of effect of intervention compared with “no treatment” for glycated hemoglobin. HbA1c, glycated hemoglobin; CI, confidence interval; N, number of participants; SD, standard deviation

Data from thirteen trials reporting the impact of the interventions on HbA1c [29-41,43,45-47] were pooled in a meta-analysis (Figure 2). The trials included thirteen comparisons assessing the impact of unidirectional and bidirectional messages. The weighted HbA1c mean difference between intervention (n=583) and control group (n=572) was -0.53% (95% CI -0.59% to -0.47%). There was no observed heterogeneity in HbA1c among the trials (*I* ^2^=0%). Very similar effects were produced by unidirectional (-0.53%, 95% CI -0.60% to -0.47%) compared with bidirectional messages (-0.52%, 95% CI -0.69% to -0.34%).

A second meta-analysis examined the impact of the interventions on BMI. Five trials [36,37,43,45,47] with a total of 406 participants were included. The BMI mean difference between the intervention and control group was -0.25 kg/m^2^(-1.02 to 0.52) and not statistically significant (Multimedia Appendix 7). There was no observed heterogeneity among the trials (*I* ^2^=0%). Unidirectional messages produced a smaller effect than bidirectional messages (0.08 [-1.76 to 1.93] vs -0.32 [-1.16 to 0.53], respectively), but the difference was not statistically significant. For both meta-analyses, sensitivity analyses confirmed that the overall results were robust to the use of imputed correlation coefficients, and Egger and Begg tests indicated an absence of publication bias.

Other clinical outcomes were too heterogeneous to pool. Unidirectional messaging interventions led to significant reductions of blood pressure in one of the two trials examining this outcome [31,37], and in one [47] of the four bidirectional message-based trials [38-40,45,47]. Improvement in lipid levels was reported for two [29,37] out of three unidirectional messaging interventions, and for three [36,39,40,47] out of four bidirectional messaging interventions. Patient-reported outcomes (diabetes-related distress, diabetes symptoms, and depression) did not significantly improve in either of the two trials that examined these parameters [30,32,39,40].

### Differences in Impact Between High-Income and Low- and Middle-Income Countries

The proportion of studies reporting positive effects was consistently higher for trials carried out in LMIC compared to HIC in all domains examined, including acceptability of the interventions (100% in LMIC vs 57% in HIC), impact on determinants of behavior-change (100% vs 0%), on physical activity and diet (67% vs 0%), and on clinical outcomes (100% vs 55%, see Multimedia Appendix 8). Subgroup meta-analysis showed a similar reduction in HbA1c in HIC (-0.53 [-0.60 to -0.47]) compared to LMIC (-0.53 [-0.69 to -0.37]).

### Use of Theory in Included Studies

The extent to which the trials explicitly used theory in relation to a number of criteria is reported in Multimedia Appendix 9. In general, theory was not used extensively. Only three trials [35,38-41] explicitly reported that the interventions were based on theory. Two interventions [35,39-41] were based on the transtheoretical model of behavioral change [48] (one study [35,41] in conjunction with the behavioral learning theory [49]), whereas the remaining intervention [38] was based on the information-motivation-behavioral skills model [50,51].

Where theory was explicitly mentioned, two trials [35,38,41] used theory to develop the intervention techniques. Only one study [38] mentioned the targeted construct that the intervention was hypothesized to change, and linked the theoretical constructs to at least one intervention technique. None of the trials measured theory-relevant constructs, used adequate measures of behavior-change, carried out a mediational analysis of constructs, or used their results to refine theory.

## Discussion

This systematic review identified 15 controlled trials examining the effectiveness of interventions to promote healthy eating and physical activity in people with type 2 diabetes, delivered via automated brief messaging sent to mobile devices. The interventions predominantly used SMS technology, addressed both diet and physical activity, and were not based on theoretical models of behavior-change. Our meta-analyses showed that automated brief messaging produced a clinically important and statistically significant effect on glycemic control (pooled effect on HbA1C= -0.53%, *P*<0.001), but not on weight loss (BMI= -0.25 kg/m^2^, *P*=0.53). In general, interventions based on the use of unidirectional messages produced similar effects compared to those based on bidirectional messages. Interventions conducted in LMIC generally showed a more positive impact than those conducted in HIC.

### Strengths and Limitations of the Review

This is the first systematic review specifically examining the impact of automated brief messages on self-management behavior in people with type 2 diabetes. Additional novel aspects of this review include an assessment of the extent to which interventions were based on theory, an assessment of the behavioral change techniques used, and an examination of the relative impact of this type of intervention in countries with different levels of economic development. Relevant trials were identified using a comprehensive search strategy and a large number of bibliographic sources.

In terms of limitations, our meta-analyses were restricted to glycemic control and BMI. Although we intended to conduct meta-analyses on other relevant outcomes (namely diabetes knowledge, attitudes toward diabetes self-management, and change in diet and physical activity-related behavior), these parameters were seldom measured, which represents a gap in evaluations to date. Finally, although formal tests on publication bias seemed to exclude its presence, we cannot completely rule out its existence.

### Comparison with Previous Reviews and Implications

The positive findings observed in our diabetes-specific review are consistent with findings from reviews examining a wide range of conditions. For example, a recent meta-analysis observed that SMS messages produced a small, positive, significant effect (g=0.29) on a broad range of healthy behaviors in patients with different types of long-term conditions [12]. A recent study reviewed 15 systematic reviews and meta-analyses, observing that the majority of published text-messaging interventions were effective when addressing weight loss, physical activity, smoking cessation, and medication adherence for antiretroviral therapy [52].

The estimated 0.53% reduction in HbA1c observed in our meta-analysis is clinically important, as evidence suggests that every percentage point decrease in HbA1c over 10 years is associated with a risk reduction of 21% for deaths related to diabetes, 14% for myocardial infarctions, and 37% for microvascular complications [53]. Our result is consistent with findings from a previous systematic review of computer-based interventions to improve diabetes self-management, which showed that interventions based on the use of mobile phones (although not specifically text messages) produced the largest HbA1c reductions (-0.5%) [19]. We deliberately focused this review on interventions to improve physical activity and healthy diet. Medication adherence is also a key aspect of diabetes self-management, and adherence behavior can also be targeted by messaging interventions. We examined the impact of brief messages to improve adherence to diabetes medication in a separate systematic review, which provided evidence that messages produced a moderate positive effect on medication adherence and clinical outcomes [17].

Interventions based on the use of one-way messages produced a very similar effect to those based on two-way messages, which can be more tailored and usually require more complex technology, and are more resource intensive. This similarity is also consistent with findings from a recent trial evaluating text messages to improve treatment adherence in people with hypertension. This result is also consistent with findings from our systematic review on messages to improve adherence to diabetes medication, which observed that interventions exclusively based on brief messages produced a similar effect compared to more complex interventions combining messaging with monitoring strategies [17].

More than half of the trials did not include measures of behavior-change, and those that did reported mixed results. Previous systematic reviews also reported mixed results. For example, Cassimatis et al [54] observed that only five out of eight trials examining the effects of type 2 diabetes behavioral telehealth interventions showed significant improvements in dietary adherence and physical activity. Cotter et al [55] observed that only two out of nine studies based on Internet interventions to support lifestyle modification for type 2 diabetes management demonstrated improvements in diet or physical activity.

Although we did not observe a statistically significant reduction in BMI, we cannot exclude a small reduction. Since the text messages specifically targeted diet and physical activity behavior, we expected a greater effect. However, there were only a low number of trials reporting BMI as an outcome. Evidence from previous systematic reviews examining the impact of text messages on weight reduction is mixed, with some suggesting lack of consistent effects [56,57] and others reporting significant weight loss [58].

The interventions seem to have been acceptable to the recipients. There are many features related to mobile technology that may engage patients with the intervention. Some of these features include ease of use, convenience (eg messages need not be retrieved immediately), mobility (eg read at home or away), and frequent reinforcement (eg can read more than once). However, measures of acceptance and usability of the interventions assessed in the review were not obtained using a validated tool.

Our results suggested that the interventions were more effective in LMIC than in HIC. As far as we know, this is the first study comparing the impact of automated brief messages on long-term condition self-management between countries with different levels of economic development. Two recent reviews evaluated the impact of mHealth interventions in patients with long-term conditions living in LMICs, concluding that they are cost-effective and can produce a positive impact on clinical outcomes, health-related quality of life [59], and treatment adherence [60]. Increasing evidence suggests that mHealth interventions are a useful tool to address health care system constraints in developing countries, namely limited health care workforce, limited financial resources, high burden of disease, and difficulties in providing health care to hard-to-reach populations [61]. These factors may partially explain the more positive impact in LMIC observed in our review.

### Limitations of Available Evidence and Future Research Needs

The studies in our review consistently supported the use of brief messages to promote healthier lifestyle behavior in patients with type 2 diabetes. However, available evidence is limited by several factors. First, most of the trials presented moderate or high risk of bias, mainly due to small sample sizes and inadequate blinding. To confirm the positive findings observed in our review, methodologically robust trials of greater size are very much needed. Second, although all the interventions specifically aimed to improve lifestyle behavior, behavior-change was measured in less than half of the studies. Where measured, a wide range of instruments were used, most having been designed *ad hoc* and not meeting adequate standards for validity or reliability. Third, only a small fraction of trials reported use of explicit behavior-change theory. Where it was mentioned, theory was used to design the intervention, but not to examine process measures that might indicate effect, or to subsequently refine theory. There is a current debate about whether or not the evidence base for behavior-change interventions can be enhanced by applying relevant theory. It has been suggested that doing so may focus attention on the mechanisms by which interventions are effective [62]. Finally, interventions used a relatively narrow range of behavior-change techniques, focusing on provision of information. Techniques such as those involving goal-setting and planning how to enact behavior or elicit social support were seldom considered, despite evidence that such techniques are generally effective at increasing physical activity in people with diabetes or obese people [63,64].

Additional research needs include an estimation of the cost-effectiveness of the interventions, an examination of their long-term impact, an understanding of what circumstances are effective (which features of the underlying health system and target population are helpful, and which features mitigate against them working), assessment of intervention safety, and an examination of their potential contribution to more comprehensive, multifaceted interventions [52].

### Conclusions

Interventions based on the use of automated brief messages sent to mobile devices to promote lifestyle behavior can improve glycemic control in patients with type 2 diabetes, both in developed and developing countries. Larger and methodologically robust trials are needed to confirm these positive findings.

## Appendix

Multimedia Appendix 1 Bibliographic searches - registry of searches.

Multimedia Appendix 2 Bibliographic searches - search strategy (Medline).

Multimedia Appendix 3 Characteristics of the identified trials and interventions.

Multimedia Appendix 4 Cochrane summary risk of bias for the included trials (n=15).

Multimedia Appendix 5 Cochrane individual risk of bias for the included trials (n=15).

Multimedia Appendix 6 Main results of the studies identified.

Multimedia Appendix 7 Weighted mean difference in size of effect of intervention compared with “no treatment” for body mass index.

Multimedia Appendix 8 Differences between low- and middle- income countries and high-income countries in the impact of the interventions.

Multimedia Appendix 9 Degree of use of theory in the development of the interventions.

## Abbreviations

BMI: body mass index
HbA1c: glycated hemoglobin
HIC: high-income country
LMIC: low- and middle-income countries
mHealth: mobile health
PRISMA: Preferred Reporting Items for Systematic Reviews and Meta-Analyses
RCT: randomized controlled trial
SD: standard deviation
SMS: short message service
