# Supplementary material for: The Impact of Automated Brief Messages Promoting Lifestyle Changes Delivered Via Mobile Devices to People with Type 2 Diabetes: A Systematic Literature Review and Meta-Analysis of Controlled Trials
Source: J Med Internet Res. 2016 Apr 19;18(4):e86. doi: 10.2196/jmir.5425 (PMC4873307; doi:10.2196/jmir.5425)
Supplement: Multimedia Appendix 1 [file jmir_v18i4e86_app1.pdf]

**Multimedia Appendix 1.** Bibliographic searches - registry of searches.

| Database | Provider | Period searched | # hits |
|---|---|---|---|
| Ovid MEDLINE(R) 1946 to April Week 3 2015 | OvidSP | 1946 to 21-Apr-15 | 852 |
| Embase 1974 to 2015 April 20 | OvidSP | 1974 to 21-Apr-15 | 1478 |
| PsycINFO 1967 to April Week 2 2015 | OvidSP | 1967 to 21-Apr-15 | 168 |
| CINAHL | EBSCOHost | 1960 to 21-Apr-15 | 282 |
| WoK (Web of Science Core Collection) | Web of Science, Thomson Reuters | 1945 to 20-Apr-15 | 891 |
| Cochrane Central Register of Controlled Trials | Cochrane Library via Wiley | Inception (unknown year) to 20-Apr-15 | 795 |
| AIM (AFRO) | World Health Organization | Inception (unknown year) to 20-Apr-15 | 1 |
| LILACS | World Health Organization | Inception (unknown year) to 20-Apr-15 | 9 |
| IMEMR | World Health Organization | Inception (unknown year) to 20-Apr-15 | 2 |
| IMSEAR | World Health Organization | Inception (unknown year) to 20-Apr-15 | 0 |
| WHOLIS | World Health Organization | Inception (unknown year) to 20-Apr-15 | 0 |
| WPRIM | World Health Organization | Inception (unknown year) to 2-Apr-15 | 5 |
| Total | | | 4483 |
| Duplicates | | | 2413 |
| **Total (unique references)** | | | **2070** |
