# Supplementary material for: The Impact of Automated Brief Messages Promoting Lifestyle Changes Delivered Via Mobile Devices to People with Type 2 Diabetes: A Systematic Literature Review and Meta-Analysis of Controlled Trials
Source: J Med Internet Res. 2016 Apr 19;18(4):e86. doi: 10.2196/jmir.5425 (PMC4873307; doi:10.2196/jmir.5425)
Supplement: Multimedia Appendix 2 [file jmir_v18i4e86_app2.pdf]

Multimedia Appendix 2. Bibliographic searches - search strategy (Medline).

	Searches	Results
1	*Diabetes mellitus/	63542
2	diabet*.ti.	228757
3	exp Diabetes Mellitus, Type 2/	90273
4	((type 2 or type ii) adj2 diabet*).ti,ab.	75968
5	((non insulin* depend* or non insulin* depend* or non-insulin?depend* or non insulin?depend*) adj2 diabet*).ti,ab.	9779
6	((matur* onset or adult onset) adj2 diabet*).ti,ab.	2100
7	(T2DM or T2D or TIIDM or TIID or NIDDM or MODY or MODM or AODM).ti,ab.	16122
8	((obes* or overweight) adj5 diabet*).ti,ab.	25239
9	1 or 2 or 3 or 4 or 5 or 6 or 7 or 8	292605
10	Telemedicine/ or (mobile health or mhealth* or m-health or telehealth or tele-health or ehealth* or e-health* or (electronic adj2 health) or telemedicine).ti,ab.	19606
11	cell phones/ or (phone* or telephone* or smartphone* or smart-phone* or iphone* or i-phone*).ti,ab. or computers, handheld/ or (((computer* or device* or technolog* or app*) adj4 (handheld* or hand-held* or palm* or mobile* or tablet* or pocket* or portable*)) or (PDA* or (Personal adj digital adj assistant*))).ti,ab.	77418
12	text messaging/ or (((text* or short* or automat* or electron*) adj6 messag*) or sms).ti,ab. or Electronic mail/ or (email* or e-mail*).ab,ti. or ((internet* or www* or web* or computer* or net* or online* or on-line* or electronic* or automat*) adj5 (messag* or communicat*)).ti,ab.	18079
13	10 or 11 or 12	109300
14	9 and 13	2193
15	randomized controlled trial.pt.	390929
16	controlled clinical trial.pt.	89138
17	((cluster* or crossover* or quasiexperiment* or quasi-experiment* or control*) adj5 (trial* or study* or design*)).ti,ab.	357759
18	(random* or (control* adj2 group*)).ti,ab.	914639
19	trial*.ab.	537576
20	15 or 16 or 17 or 18 or 19	1476609
21	exp animals/ not humans.sh.	4023388
22	(rat or rats or rodent? or mice or mouse or cow or cows or cattle or calf or calves or ewe? or sheep or goat or ruminant? or chicken? or horse or horses or murine or bovine or ovine or animal?).ti.	1491367
23	20 not (21 or 22)	1259655
24	14 and 23	**852**
