# Supplementary material for: The Impact of Automated Brief Messages Promoting Lifestyle Changes Delivered Via Mobile Devices to People with Type 2 Diabetes: A Systematic Literature Review and Meta-Analysis of Controlled Trials
Source: J Med Internet Res. 2016 Apr 19;18(4):e86. doi: 10.2196/jmir.5425 (PMC4873307; doi:10.2196/jmir.5425)
Supplement: Multimedia Appendix 3 [file jmir_v18i4e86_app3.pdf]

Multimedia Appendix 3. Characteristics of the identified trials and interventions.

Reference/ Country/ Setting	Study design/ Comparator/ Duration (months)	Messages (Type/ Format/ Content)	Messages (Frequency[a]/ Timing[b])	Mobile devices and applications	Use of theory	Behavior change techniques
Tsang et al. 2001 [44]<br>China<br>Hospital care	Cross over<br>Usual care<br>6	Bidirectional<br>Image (graphs)<br>Diet	2 per week/<br>Not reported	Hand-held electronic diary with a touch-screen (CV8300, Vtech, Hong Kong)	Not reported	Provide feedback on performance
Yoo et al. 2009 [47]<br>South Korea<br>University and community healthcare center	RCT<br>Usual care<br>3	Bidirectional<br>Text (SMS)<br>Physical activity+ diet + other	3 per day/<br>Not reported	Mobile phone (LG-SV280; LGElectronics, Seoul, Korea)	Not reported	Provide information on consequences;<br>Provide instruction;<br>Provide feedback on performance;<br>Provide contingent rewards;<br>Prompt practice;<br>Time management
Noh et al. 2010 [37]<br>South Korea<br>Hospital care	RCT<br>Minimal intervention<br>7	Unidirectional<br>Text (website)<br>Physical activity+ diet + other	Not reported	Web-based ubiquitous information system (SK telecom [Seoul, Republic of Korea]).<br>All mobile phones using the International Mobile Telecommunication-2000 system could connect to the system.	Not reported	Stress management
Lim et al. 2011 [36]<br>South Korea<br>Hospital care	RCT<br>Usual care<br>6	Bidirectional<br>Text (SMS)<br>Physical activity+ diet + other	Variable (at least 8 per week)/<br>Not reported	Glucometers specifically devised for ubiquitous healthcare service (GlucoDr Supersensor, AGM-2200, Allmedicus, Korea).The glucometer transferred the tested data and stored it in a remote server.	Not reported	Provide feedback on performance;<br>Prompt practice
Quinn et al. 2011 [39,40]<br>US<br>Primary care	Cluster RCT<br>Usual care<br>12	Bidirectional<br>Text (SMS)<br>Physical activity+ diet + other	Variable (depending on patients' needs)/<br>Not reported	One Touch Ultra 2 glucose meter (LifeScan, Milpitas, CA), mobile phones, and a diabetes management software.	Trans-theoretical model	Provide instruction;<br>Provide feedback on performance
Shetty et al.	RCT	Unidirectional	2 per week/	Mobile phone	Not reported	Provide information

2011 [42]<br>India<br>Primary care	Usual care<br>12	Text (SMS)<br>Physical activity+ diet + other	Not reported			on consequences;<br>Prompt self-monitoring of behavior;<br>Prompt practice
Bell et al. 2012 [31]<br>US<br>Specialized care	RCT<br>Usual care<br>12	Unidirectional<br>Video<br>Physical activity+ diet + other	1 per day/<br>Variable (participants were allowed to view the video multiple times throughout the 24-hour period before the next video was sent)	Broadband-enabled cell phone	Not reported	Provide information about behavior-health link;<br>Provide information on consequences;<br>Provide instruction;<br>Stress management;<br>Time management
Goodarzi et al. 2012 [34]<br>Iran<br>Community	RCT<br>Usual care<br>3	Unidirectional<br>Text (SMS)<br>Physical activity+ diet + other	4 per week/<br>Not reported	Patients' mobile phone	Not reported	Provide information on consequences;<br>Prompt intention formation;<br>Provide instruction
Abebe et al. 2013 [29]/<br>Capozza et al. 2015 [33]<br>US<br>Primary care	RCT<br>Usual care<br>6	Unidirectional<br>Text (SMS)<br>Physical activity+ diet + other	Variable (between one and seven messages per day, depending on participants' preference)/<br>Sent at convenient timing for participants	Patients' mobile phone	Not reported	Prompt intention formation;<br>Prompt self-monitoring of behavior;<br>Provide feedback on performance;<br>Provide contingent rewards;<br>Prompt practice;<br>Stress management
Orsama et al. 2013 [38]<br>Finland<br>Community	RCT<br>Usual care<br>10	Bidirectional<br>Text (SMS)<br>Physical activity+ diet + other	Not reported	Mobile telephone, software application, and assessment devices	Information-motivation-behavioral skills model	Provide information on consequences;<br>Provide instruction;<br>Provide feedback on performance
Arora et al. 2014 [30]/<br>Burner et al. 2014 [32]<br>US<br>Hospital care	RCT<br>Usual care<br>6	Unidirectional<br>Text (SMS)<br>Physical activity+ diet + other	2 per day/<br>9 am and 5pm	Patients' mobile phone	Not reported	Provide information on consequences;<br>Prompt intention formation;<br>Provide instruction
Tamban et al. 2014 [43]<br>Philippines<br>Unclear	RCT<br>Usual care<br>6	Unidirectional<br>Text (SMS)<br>Physical activity+ diet + other	3 per week/<br>Sent at convenient timing for participants	Patients' mobile phone	Not reported	Provide information about behavior-health link;<br>Provide information on consequences;<br>Provide instruction;

						Prompt specific goal setting; Prompt self-monitoring of behavior; Provide feedback on performance
Islam et al. 2014 [35]/ Islam et al. 2015 [41] Bangladesh Hospital care	RCT Usual care 6	Unidirectional Text (SMS) Physical activity+ diet + other	1 per day/ Not reported	Patients' mobile phone	Behavioral learning theory and trans-theoretical model of behavioral change	Provide information about behavior-health link; Provide information on consequences; Provide instruction; Stress management
Waki et al. 2014 [45] Japan Hospital care	RCT Usual care 3	Bidirectional Text (SMS) Physical activity+ diet	Not reported	Smartphone (NEC, Tokyo, Japan: MEDIAS WP N-06C), NFC-enabled glucometer (Terumo, Tokyo, Japan: MS-FR201B), BP monitor (Omron, Kyoto, Japan: HEM- 7081-IT), pedometer (Omron HJ-720IT), and scale (Omron HBF-206IT).	Not reported	Prompt barrier identification; Provide feedback on performance
Yarahmadi et al. 2014 [46] Iran Specialized care	RCT Unclear 2	Unidirectional Text (SMS) Physical activity+ diet + other	3 per week/ Not reported	Patients' mobile phone	Not reported	Unclear

a: Number of messages sent per week.
b. Time of the day when the messages were sent.
RCT, randomized controlled trial; SMS, short message service;
