# Supplementary material for: The Impact of Automated Brief Messages Promoting Lifestyle Changes Delivered Via Mobile Devices to People with Type 2 Diabetes: A Systematic Literature Review and Meta-Analysis of Controlled Trials
Source: J Med Internet Res. 2016 Apr 19;18(4):e86. doi: 10.2196/jmir.5425 (PMC4873307; doi:10.2196/jmir.5425)
Supplement: Multimedia Appendix 4 [file jmir_v18i4e86_app4.pdf]

Multimedia Appendix 4. Cochrane summary risk of bias for the included trials (n = 15).
