# Supplementary material for: The Impact of Automated Brief Messages Promoting Lifestyle Changes Delivered Via Mobile Devices to People with Type 2 Diabetes: A Systematic Literature Review and Meta-Analysis of Controlled Trials
Source: J Med Internet Res. 2016 Apr 19;18(4):e86. doi: 10.2196/jmir.5425 (PMC4873307; doi:10.2196/jmir.5425)
Supplement: Multimedia Appendix 5 [file jmir_v18i4e86_app5.pdf]

Multimedia Appendix 5. Cochrane individual risk of bias for the included trials (n =15)

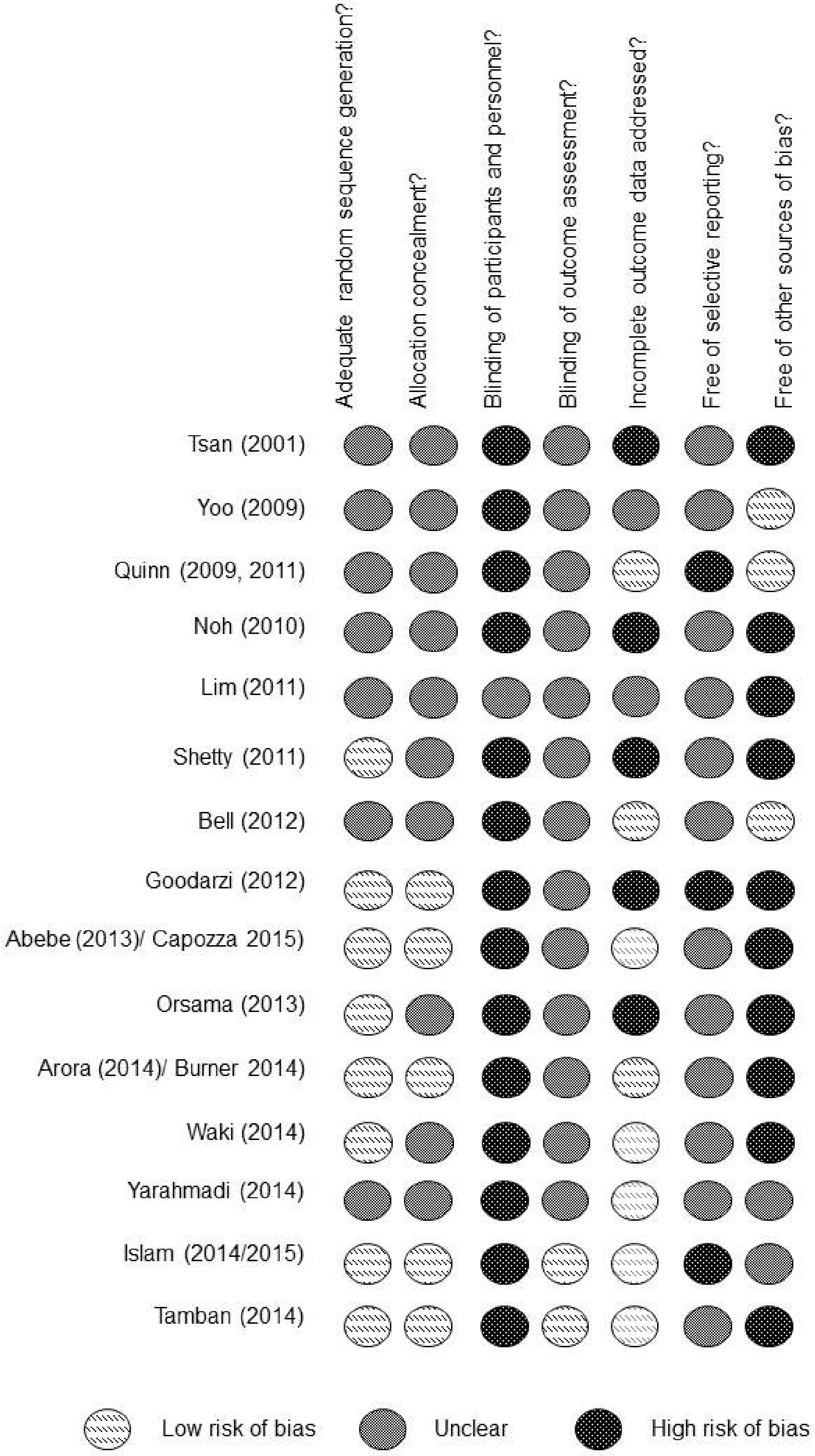

Adequate random sequence generation?
Allocation concealment?
Blinding of participants and personnel?
Blinding of outcome assessment?
Incomplete outcome data addressed?
Free of selective reporting?
Free of other sources of bias?
Tsan (2001)
Yoo (2009)
Quinn (2009, 2011)
Noh (2010)
Lim (2011)
Shetty (2011)
Bell (2012)
Goodarzi (2012)
Abebe (2013)/ Capozza 2015)
Orsama (2013)
Arora (2014)/ Burner 2014)
Waki (2014)
Yarahmadi (2014)
Islam (2014/2015)
Tamban (2014)
Low risk of bias
Unclear
High risk of bias
