# Supplementary material for: The Impact of Automated Brief Messages Promoting Lifestyle Changes Delivered Via Mobile Devices to People with Type 2 Diabetes: A Systematic Literature Review and Meta-Analysis of Controlled Trials
Source: J Med Internet Res. 2016 Apr 19;18(4):e86. doi: 10.2196/jmir.5425 (PMC4873307; doi:10.2196/jmir.5425)
Supplement: Multimedia Appendix 6 [file jmir_v18i4e86_app6.pdf]

Multimedia Appendix 6. Main results of the studies identified.

Reference/ Type of messages	Acceptability	Knowledge/ Attitudes/ Self-efficacy	Behavior change (diet/physical activity)	Clinical and patient reported outcomes
Noh et al. 2010 [37]/ Unidirectional	Low acceptability due to the lack of a user-friendly interface and inexperience with mobile web use	Not reported	Not reported	Glycemic control: No statistical difference in between groups at 6 months (7.5% ±0.4% vs. 8.1± 0.3%). Lipids: Statistically significant improvement in LDL-cholesterol (but not in TG or HDL-cholesterol) in the intervention group, whereas no differences were observed in the control group. Blood pressure: No statistically significant improvement in the intervention group. Weight: No differences observed
Shetty et al. 2011 [42] /Unidirectional	Highly acceptable to the patients, as seen from the number of messages and their frequency requested by the patients	Not reported	Physical activity: Marginal improvement (not statistically significant) in the intervention group. Diet: No statistically significant changes	Glycemic control: The proportion of patients with HbA1c<8% significantly increased in the intervention group (from 30.8% to 55.1%) whereas not changes were observed in the control group. Lipids: No differences observed in total and LDL-cholesterol. BMI: No differences observed
Bell et al. 2012 [31] / Unidirectional	11 participants did not view videos at all or did it briefly at the beginning of their participation and then stopped in the first 2 months; 2 participants viewed the videos throughout the active intervention but <10/month; 10 participants viewed more than 10 videos/month	Not reported	Not reported	Glycemic control: No statistically significant differences observed. Blood pressure: No differences observed. Weight: No differences observed
Goodarzi et al. 2012 [34] / Unidirectional	Not reported	Knowledge: significant improvement/ Attitudes: no significant improvement/ Self-efficacy: significant improvement	Physical activity: statistically significant improvement in the intervention group. Diet: statistically significant improvement in the intervention group	Glycemic control: significant improvement in HbA1C for the experimental group. Lipids: significant change in cholesterol for the experimental group but not in LDL, triglycerides, or HDL.
Abebe et al. 2013 [29] & Capozza et al. 2015 [33] / Unidirectional	High satisfaction. Moderate usability (40% of the participants requested stop receiving the messages before the end of the intervention)	Not reported	Not reported	Glycemic control: No statistically significant differences between the intervention and control groups
Arora et al. 2014	Subjects rated satisfaction	Knowledge: Not	Physical activity: No	Glycemic control: The primary

[30] & Burner et al. 2014 [32] / Unidirectional	with the TExT-MED program very highly. No patients opted out of the program	statistically significant improvement in the intervention group (mean=-0.7 (95%CI= -1.5 to 0.1))/ Self-efficacy: Not statistically significant improvement in the intervention group (mean=0.1 (95%CI= 0.2 to 0.4))	statistically significant effect observed. Diet: No statistically significant effect observed.	outcome of median HbA1c decreased by 1.05% in the TExT-MED group compared with 0.60% in the control group (difference not statistically significant). Patient reported outcomes: No statistically significant improvement in diabetes related emotional distress (PAID questionnaire) in the intervention group
Tamban et al. 2014 [43] / Unidirectional	Not reported	Not reported	Physical activity: Statistically significant improvement in physical activity (number of minutes) is seen after 6 months, favoring the SMS group. Diet: statistically significant improvement in the intervention group	Glycemic control: Statistically significant reduction in HbA1c in the intervention (-0.82%) when compared to the control group (-0.52%). BMI: significant reduction in the intervention when compared with control group
Islam et al. 2014 [35]/ Islam et al. 2015 [41]/ Unidirectional	Not reported	Not reported	Not reported	Glycemic control: Statistically significant HbA1c reduction in the intervention (-0.82%) when compared to the control group (0.18%)
Yarahmadi et al. 2014 [46] / Unidirectional	Not reported	Not reported	Not reported	Glycemic control: Higher reduction in HbA1c in the intervention (-0.65%) when compared to the control group (-0.12%)
Tsang et al. 2001 [44] / Bidirectional	Acceptability: 95% patients found the device easy to operate while 63% found it useful	Not reported	Diet: 35% of the patients were consuming the recommended carbohydrate portions as stated in their meal plan. 60% had a tendency to over-consume, 5% under-consume	Glycemic control: Use of the system was associated with a significant reduction of HbA1c concentration compared with the control period (mean HbA1c difference between control and intervention group = -0.83%)
Yoo et al. 2009 [47] / Bidirectional	Participants did not find the system difficult to use. They were satisfied with the continuous care of their chronic disease	Not reported	Not reported	Glycemic control: significant improvements in HbA1c in the intervention group (-0.5%) compared with the control group (0.1%). Lipids: Total cholesterol and LDL-cholesterol levels were significantly decreased after 3 months in the intervention group

				compared with the control group<br>Blood pressure: Significant reduction in the systolic and diastolic blood pressure only in the intervention group.<br>Weight and waist circumference: No differences observed
Lim et al. 2011 [36] / Bidirectional	Not reported	Not reported	Not reported	Glycemic control: The proportion of patients that achieved HbA1c<7.0% without hypoglycemia (primary end point of the study) was significantly higher in the intervention than in the control group (30.6% vs 14.0%).<br>Lipids: significant reduction in LDL-cholesterol in the intervention group when compared to the control group.<br>Weight and BMI: significant reduction in the u-healthcare group compared with control group
Quinn et al. 2011 [39,40] / Bidirectional	Not reported	Not reported	Not reported	Glycemic control: significant reduction of HbA1c in the intervention group (-1.9% in comparison with the control group (-0.7%).<br>Lipids: Reduction in the intervention group in total cholesterol, triglycerides, HDL-cholesterol, and LDL-cholesterol, but differences were only statistically significant for total cholesterol.<br>Blood pressure: No statistically significant differences.<br>Patient reported outcomes: No significant differences in "Diabetes Distress Scale", "Diabetes Symptom Inventory", and depression (PHQ-9)
Orsama et al. 2013 [38] / Bidirectional	100% of intervention participants regarded the mobile telephone application, as ‘‘very easy’’ or ‘‘quite easy’’ to use. More than 90% reported that making health parameter measurements and reporting them was ‘‘very useful’’ or ‘‘quite useful,’’ and approximately 82% regarded the automatic feedback they received as ‘‘very useful’’ or ‘‘quite useful.’’	Not reported	Not reported	Glycemic control: Intervention participants achieved, compared with controls, a significantly greater mean reduction in HbA1c (-0.40% vs 0.04%).<br>Blood pressure: No statistically significant improvement in the intervention group.<br>Weight: significant reduction in the u-healthcare group (-2.1 kg) compared with control group (-0.4 kg)
Waki et al. 2014	Usability: participants were	Not reported	Physical activity: No	Glycemic control: HbA1c

[45] / Bidirectional	comfortable with the use of the equipment.		statistically significant effect observed/<br>Diet: No statistically significant effect observed	decreased an average of 0.4% compared with an average increase of 0.1% in the control group (statistically significant differences observed).<br>Lipids: no differences observed in LDL, HDL and TG.<br>Blood pressure: No differences observed<br>BMI: significant reduction in the intervention when compared with control group

BMI, body mass index; CI, confidence interval; HbA1c, glycemic hemoglobin; HDL, high density lipoprotein; LDL, low density lipoprotein; PHQ, Patient Health Questionnaire; TG, triglycerides.
