# Supplementary material for: The Impact of Automated Brief Messages Promoting Lifestyle Changes Delivered Via Mobile Devices to People with Type 2 Diabetes: A Systematic Literature Review and Meta-Analysis of Controlled Trials
Source: J Med Internet Res. 2016 Apr 19;18(4):e86. doi: 10.2196/jmir.5425 (PMC4873307; doi:10.2196/jmir.5425)
Supplement: Multimedia Appendix 7 [file jmir_v18i4e86_app7.pdf]

Multimedia Appendix 7. Weighted mean difference in size of effect of intervention compared with “no treatment” for body mass index.

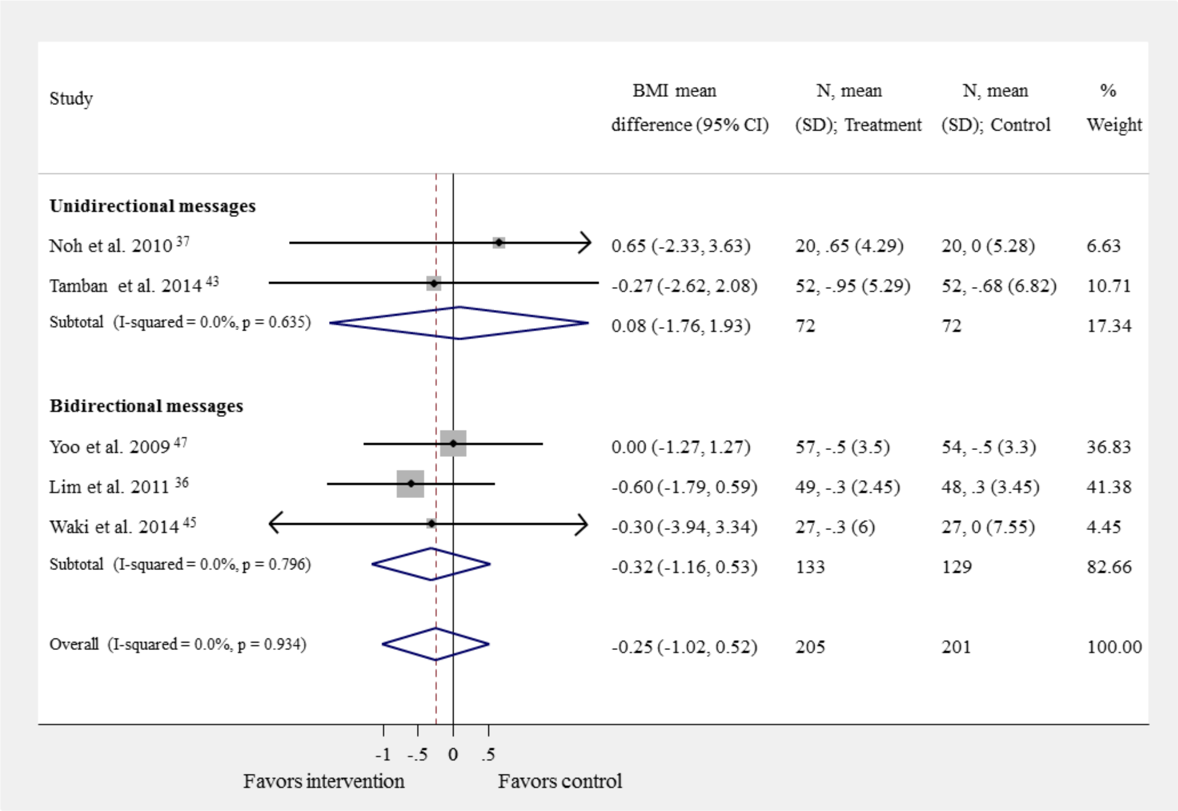

BMI, body mass index; CI, confidence interval; N, number of participants; SD, standard deviation.
