# Supplementary material for: The Impact of Automated Brief Messages Promoting Lifestyle Changes Delivered Via Mobile Devices to People with Type 2 Diabetes: A Systematic Literature Review and Meta-Analysis of Controlled Trials
Source: J Med Internet Res. 2016 Apr 19;18(4):e86. doi: 10.2196/jmir.5425 (PMC4873307; doi:10.2196/jmir.5425)
Supplement: Multimedia Appendix 8 [file jmir_v18i4e86_app8.pdf]

**Multimedia Appendix 8.** Differences between low- and middle- income countries and high income countries in the impact of the interventions.

HIC, high income country; LMIC, low and middle income country.
"Positive impact" indicates statistically significant improvement of the outcomes of interest in the intervention group in comparison with the control group.
"No impact" indicates no statistically significant improvement observed.
