# Supplementary material for: The Impact of Automated Brief Messages Promoting Lifestyle Changes Delivered Via Mobile Devices to People with Type 2 Diabetes: A Systematic Literature Review and Meta-Analysis of Controlled Trials
Source: J Med Internet Res. 2016 Apr 19;18(4):e86. doi: 10.2196/jmir.5425 (PMC4873307; doi:10.2196/jmir.5425)
Supplement: Multimedia Appendix 9 [file jmir_v18i4e86_app9.pdf]

Multimedia Appendix 9. Degree of use of theory in the development of the interventions*

Criterion	Description	Number of trials for which there is some evidence that this criterion is satisfied
1. Theory/model of behavior mentioned	Models/theories that specify relations among variables, in order to *explain* or *predict* behavior are mentioned, even if the intervention is not based on this theory.	3[35,38-41]
2. Targeted construct mentioned as predictor of behavior	Evidence that the 'targeted' construct that the study intervention is hypothesized to change is presented within the introduction or method	1[38]
3. Intervention based on single theory	The intervention is based on a single theory (rather than a combination of theories or theory predictors).	2[35,38,41]
4. Theory used to select recipients for the intervention	Participants were screened/selected based on achieving a particular score/level on a theory-relevant construct.	0
5. Theory used to select/ develop intervention techniques	The intervention is explicitly based on a theory or combination of theories and predictors.	2 [35,38,41]
6. Theory used to tailor intervention techniques to recipients	The intervention differs for different sub-groups that vary on a psychological construct (e.g., stage of change) at baseline.	0
7. All intervention techniques are explicitly linked to at least one construct	Each intervention technique is explicitly linked to at least one theory-relevant construct.	0
8. At least one, but not all, of the intervention techniques are explicitly linked to at least one theory-relevant construct	At least one, but not all, of the intervention techniques are explicitly linked to at least one theory-relevant construct.	1[38]
9. Group of techniques are linked to a group of constructs	A cluster of techniques is linked to a cluster of constructs.	0
10. All theory-relevant constructs are explicitly linked to at least one intervention technique	Every theoretical construct within a stated theory (see item 5) is linked to at least one intervention technique.	0
11. At least one, but not all, of the theory relevant constructs are explicitly linked to at least one intervention technique	At least one, but not all, of the theoretical constructs within a stated theory are linked to at least one intervention technique.	1[38]
12. Theory-relevant constructs are measured	(a) At least one construct of theory mentioned in relation to the intervention is measured post-intervention.	0

13.	Quality of measures	At least one of the measures of theory relevant constructs had some evidence for their reliability.<br><br>At least one of the measures of theory relevant constructs have been previously validated<br><br>The behavior measure had some evidence for its reliability<br><br>The behavior measure has been previously validated.	0
17.	Randomization of participants to condition	Do the authors claim randomization?<br><br>Is a method of random allocation to condition described (e.g., random number generator; coin toss).<br><br>Was the success of randomization tested?<br><br>Was the randomization successful (or baseline differences between intervention and control group statistically controlled)?	3[35,38-41]
21.	Changes in measured theory-relevant constructs	The intervention leads to significant change in at least one theory-relevant construct/ predictor (vs. control group) in favor of the intervention.	0
22.	Mediational analysis of construct(s)	Any evidence of hypothesized mediating variable or change in hypothesized mediating variable predicting dependent variable?	0
23.	Results discussed in relation to theory	Results are discussed in terms of the theoretical basis of the intervention	1[38]
24.	Appropriate support for theory	Support for the theory is based on appropriate mediation OR refutation of the theory is based on obtaining appropriate null effects	0
25.	Results used to refine theory	The authors attempt to refine the theory upon which the intervention was based by either adding or removing constructs to the theory, or specifying that the interrelationships between the theoretical constructs should be changed	0

* Extent to which the interventions are theory-based, examined by using the theory coding scheme developed by Michie et al[22].Information reported only for the four trials that reported the use of a theoretical model for the development of the intervention(s) (item 1 = yes).
